# A Non-Invasive Simplified Model for Estimating Lower Limb Muscle Forces During Slow Gait in Older Adults and Post-Stroke Individuals

**DOI:** 10.3390/biomimetics11040226

**Published:** 2026-03-26

**Authors:** Kun Liu, Hongxiang Guo, Jiaying Liu, Jialun He

**Affiliations:** College of Intelligent Manufacturing and Energy Engineering, Zhejiang University of Science and Technology, Hangzhou 310023, China

**Keywords:** non-invasive muscle force estimation, muscle architecture index regression, slow gait, digital twin musculoskeletal model

## Abstract

This study proposes a non-invasive, simplified muscle force estimation model (NSMFEM) designed for elderly individuals and stroke patients under slow walking conditions. The model estimates lower limb muscle forces dynamically using only kinematic parameters—with real-time muscle fiber length as the key variable—thus avoiding the limitations of traditional surface electromyography (sEMG)-based approaches such as environmental interference, signal noise, and difficulty in obtaining deep muscle sEMG. A personalized Digital Twin Musculoskeletal Model (DTMSM) was constructed by scaling a reference kinematic model and calibrating muscle origin/insertion markers based on individual anthropometry. Muscle architecture indices were derived from a multiple regression model with publicly available anatomical data. Twelve elderly subjects (eight healthy ESND and four post-stroke ESP) were evaluated at varying walking speeds. Results at slow speeds (X-slow and slow) show strong Pearson correlations between NSMFEM predictions and reference data for the majority of nine representative lower limb muscles (e.g., TFL, Iliacus, Pectineus, Tib_Ant, Soleus); passive forces of TFL, Iliacus, and Vas_Int also correlate strongly. As speed rises, correlations for some muscles (e.g., Vas_Int, Tib_Post) decline, reflecting the growing influence of segmental acceleration and muscle activation—factors omitted in the model. For stroke patient gait (ESP), Spearman analysis indicates maintained strong correlations for affected side muscles Glut_Max1, TFL, Pectineus, and Soleus, supporting the model’s utility in stroke rehabilitation assessment. Overall, NSMFEM offers a practical, sEMG free method for non-invasive dynamic muscle force estimation in slow walking elderly and post-stroke populations, aiding functional assessment and personalized rehabilitation planning. Future efforts will aim to incorporate muscle activation corrections to extend the model to faster walking speeds.

## 1. Introduction

With the increasing trend of global population aging, the incidence of stroke-induced functional dyskinesia among the elderly is rising annually [1]. Accurate muscle force estimation (MFE) is crucial for the rehabilitation of movement disorders. It underpins studies on artificial muscle function, exploration of pathological movement mechanisms, surgical planning for joint replacement, and optimization of treatment biomechanics [2]. Furthermore, understanding human neuromuscular control and accurately estimating muscle forces is crucial for clinical biomechanics and movement analysis [3]. Such knowledge not only enhances the reliability of musculoskeletal simulations but also provides essential biological insights for biomimetic system design. Neuromusculoskeletal modeling has been increasingly applied to estimate subject-specific muscle forces and to bridge the gap between human motor control and assistive device operation [4,5]. In the rapidly developing field of biologically inspired robotics and wearable exoskeletons, replicating human-like movement requires precise knowledge of how human muscles generate force under various kinematic conditions [6]. Therefore, developing an efficient and accurate muscle force estimation model is highly relevant to the design of advanced biomimetic control systems and human-machine interaction frameworks. Elderly individuals with motor dysfunction or post-stroke patients often exhibit asymmetric or compensatory gait patterns due to muscle weakness [7]. Prolonged compensatory gait can lead to severe disability, significantly impairing patients’ quality of life and limiting their mental and social participation [8]. As hemiplegia is a primary cause of gait disorders, restoring gait function remains a paramount rehabilitation goal [9]. Consequently, clinicians and researchers employ various strategies to assess walking ability and muscle power generation to correct gait asymmetry [10,11,12]. Muscle synergy is essential for maintaining dynamic balance during gait. Therefore, evaluating the balance ability of the elderly and the gait recovery in patients with motor dysfunction necessitates a primary focus on lower limb muscle forces. Crucially, recent systematic reviews on post-stroke rehabilitation, such as those examining the effects of rhythmic auditory cueing (Ghai et al., 2019), emphasize that measurable gait and postural outcomes are clinically essential for evaluating intervention efficacy beyond conventional scales [13]. However, it is important to clarify that while these reviews establish the clinical imperative for obtaining such data, they provide contextual background rather than direct methodological validation for the specific NSMFEM framework proposed in this study. Invasive measurement of muscle force is clinically impractical [14], and direct measurement of muscle parameters remains challenging [15]. While traditional surface electromyography (sEMG) is non-invasive, it suffers from limitations including susceptibility to environmental interference, signal noise, and cumbersome processing [16,17]. Although sEMG can characterize muscle activation intensity for force estimation [18], its application is constrained. Obtaining sEMG from deep muscles during gait is difficult, and its correlation with force in both deep and superficial muscles, especially during concentric/eccentric contractions, is limited. In the elderly, sEMG signals are prone to step changes [19], complicating activation calculation. In post-stroke patients, muscle reinnervation alters the sEMG-force relationship [20], with paretic muscles exhibiting changed activation patterns [21]. Furthermore, averaged sEMG may not reflect the true muscle firing relationship, necessitating advanced spatiotemporal processing methods [22], which are often impractical for long-term, real-time monitoring [23]. Real-time force estimation is also critical for human-machine interactive rehabilitation robots to determine appropriate assistive torque [24]. Another key consideration is that elderly people and stroke patients typically have slower gait speeds when walking. Fast walking protocols do not match their gait characteristics [25], and existing computational models, which heavily rely on standard static optimization, often exhibit varying predictive consistency and may not generalize well to the altered neuromuscular control of slow walking [26]. While OpenSim serves as the gold standard for musculoskeletal analysis, its application in routine clinical settings is often limited by high computational demands, complex setup procedures, and the requirement for extensive biomechanical and anatomical expertise, such as defining maximum isometric forces for specific populations, precisely tuning marker weights during scaling, and managing reserve actuators during static optimization. Consequently, there is a need for a computationally efficient, non-invasive alternative. The proposed NSMFEM is designed to address this gap by providing rapid estimation of muscle forces based on readily available kinematic data, specifically optimized for slow gait assessment, while using OpenSim as a benchmark for validation. Therefore, there is a need to explore MFE models tailored to slow walking conditions.

This paper proposes a novel, non-invasive approach to estimate lower limb muscle forces during gait in the elderly and rehabilitation patients. Based solely on human kinematics, it circumvents sEMG limitations. Kinematic data collected during slow walking is input into a personalized musculoskeletal model to calculate time-varying muscle lengths. Subsequently, active and passive muscle forces are computed using theoretical formulas [14,27]. This framework significantly reduces algorithmic complexity, providing a real-time capable solution suitable for integration into future bionic device programming and intelligent exoskeleton controllers. Results are compared with benchmarking data to validate the method’s feasibility, offering a new avenue for lower limb muscle force estimation in slow gait. Beyond technical validation, this study provides a novel comparative baseline to quantify the decoupling effect of muscle force and kinematics in post-stroke patients. This offers a meaningful mathematical perspective for evaluating pathological neuromuscular impairment, directly indicating the compensatory requirements for biomimetic rehabilitation robots to correct gait asymmetry in slow gait.

## 2. Method

It is difficult to implement a universal musculoskeletal model for non-invasively calculating muscle forces based on experimental statistics of a large number of corpses or living organisms, and targeted experimental measurement and analysis for each elderly person or patient will increase the complexity of the research, which is not user-friendly for all the experimenters. Therefore, an original solution was proposed in this paper. Based on the anatomical model of Shan, D.M. [27], the lower limb kinematic model established by Thelen was scaled [28], in which the marks of muscle origins and terminations were also recalibrated to align with the physical characteristics such as the height, weight and length of each limb segment of target elderly subjects, resulting in a personalized Digital Twin Musculoskeletal Model (DTMSM) that conforms to the physical characteristics of any target elderly person with high accuracy. In order to verify the feasibility of the proposed solution, a motion capture system was used to analyze the gait kinematic parameters of the subjects for calculating the real-time muscle fiber length of the main muscles in the lower limbs during the gait. Furthermore, based on the theory of anatomy by Delp et al. [29], a multiple regression calculation model was used to calculate the muscle architecture index. Finally, the real-time muscle fiber length and the muscle structural index were incorporated into the muscle force calculation formula by Kaufman et al. [14] to calculate the lower limb muscle forces of the specified elderly person during walking.

### 2.1. Experimental Data Collection

Twelve elderly subjects participated: eight elderly subjects with no history of lower limb disease (ESND) and four elderly stroke patients (ESP, Fugl-Meyer lower limb score: 18.5 ± 5.5) capable of independent gait. Anthropometric data (height, weight, sternum-head distance, sitting height) were recorded (Age: 65.13 ± 7.83 yrs; Height: 1642.63 ± 85.36 mm; Mass: 65.07 ± 13.94 kg). Thirty-nine reflective markers were placed for motion capture. Static calibration was performed. Kinematic data and ground reaction forces were collected using a gait platform (AMTI Inc., Watertown, MA, USA) and eight infrared cameras (Vicon, Oxford, UK). sEMG of the right leg muscles (Tibialis Anterior, Vastus Medialis, etc.) was recorded (BTS FREEEMG) after skin preparation. To analyze the effect of speed, the eight ESND performed walking tasks at four speeds: X-slow (0.61 ± 0.09 m/s), slow (0.80 ± 0.12 m/s), free (1.17 ± 0.14 m/s), and R-fast (1.64 ± 0.18 m/s) (Experiment A). The four ESP performed self-selected free walking to analyze compensatory gait (Experiment B). The study was approved by the Jilin University Ethics Committee (No. 2023-229).

### 2.2. Establishment of Digital Twin Skeleton Model (DTSM)

When establishing a DTSM that conforms to the physical characteristics of the elderly, in order to achieve accurate scaling of the skeleton model, it is necessary to collect corpse height, weight, length of each limb segment and the torso in anatomical experiments. Due to the reference [3] only providing data related to the lower limbs of the corpse without specific data of the upper limbs and torso, this paper attempts to propose a mathematical analysis method based on the comparison of the corresponding physical characteristic parameters between the subjects recruited in the experiment and the corpses provided in the literature, to complete the data of the upper limbs and torso that conforms to the physical characteristics of the elderly, and then obtain a whole-body DTSM for any designated elderly person. Based on statistical analysis of the anthropometric data in the literature [30], it is found that the sternum-head distance (*y*) is directly related to the height (*x_h_*), weight (*x_w_*), and sitting height (*x_s_*). Therefore, a regression calculation model of *y* related to *x_h_*, *x_w_*, and *x_s_* is established according to the collected limb segment parameters of all the twelve elderly subjects in the experiment and the regression calculation model was expressed by Formula (1).

(1) $$y=\left\{\begin{array}{l} 0.3674x_{h}-297.0699,\;x_{h}\\ 2.1135x_{w}-188.2610,\;x_{w}\\ 0.7111x_{s}-310.3478,\;x_{s} \end{array}\right.$$

where *r*^2^*_xh_* = 0.97, *r*^2^*_xw_* = 0.52, *r*^2^*_xs_* = 0.94, so the height *x_h_* was selected as the independent variable to finally calculate the sternum-head distance *y*. Therefore, the parameters of the torso that conform to the physical characteristics of any elderly person can be obtained. Specifically, the sternum-head distance (*y*) is utilized to scale the torso segment length and estimate the trunk’s center of mass in the whole-body kinematic model, ensuring an accurate dynamic balance calculation. Finally, based on the data applied in anatomical experiments [30] including height, weight, length of each limb segment, and the sternum-head distance calculated by the regression calculation model (1) from the height of any recruited subject, the scaled DTSM that conforms to his physical characteristics can be achieved through geometric linear scaling, which calculates scale factors based on the ratio of distances between the experimental markers on the subjects and the corresponding virtual markers on the generic model.

### 2.3. Establishment of Digital Twin Muscle Model

To estimate muscle forces without relying on sEMG, we simplified the classical Hill-type muscle model and proposed a regression approach to derive essential muscle architecture parameters.

#### 2.3.1. Calibration of the Marks of Muscle Origins and Terminations

**Figure 1:**
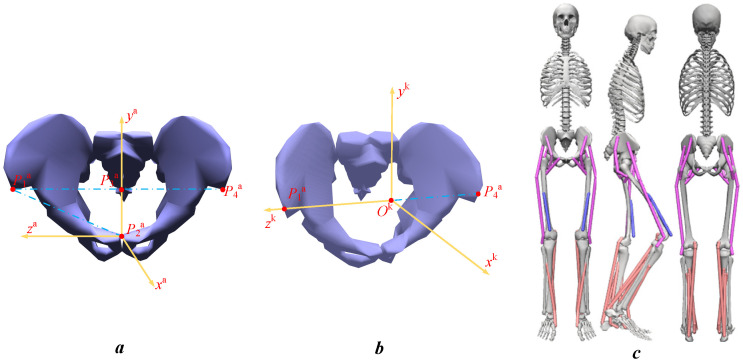
(**a**) Pelvic anatomical coordinate system. (**b**) Pelvic kinematic coordinate system. (**c**) Human Digital Twin Musculoskeletal model.

In order to reconstruct muscles for the established DTSM that conform to the physical characteristics of the elderly, it is necessary to map the marks of muscle origins and terminations in the anatomical coordinate system to the lower limb kinematic coordinate system of the DTSM. Based on the anatomical characteristics of the skeleton, anatomical coordinate systems are defined for each body segment: pelvic coordinate system, femoral coordinate system, tibial coordinate system, and foot bone coordinate system. At the same time, in order to conduct kinematic analysis, corresponding kinematic coordinate systems are established for each lower limb segment. The established anatomical coordinate system and kinematic coordinate system for the same limb segment share a same direction basis, so the transition matrix between the two coordinate systems can be solved to achieve coordinate transformation from the anatomical coordinate system to the lower limb kinematic coordinate system. Taking the pelvis as an example, in anatomical experiments, *P*_2_^a^(*x*^a^, *y*^a^, *z*^a^, 1) is defined as the anatomical pelvic coordinate system (Figure 1a), whose coordinate system origin *P*_2_^a^ is at the midpoint of the superior margin of pubic symphysis. The midpoint of the line connecting the right anterior superior iliac spine point *P*_1_^a^ and the left anterior superior iliac spine point *P*_4_^a^ is *P*_3_^a^, and the direction of the *y*^a^ axis is in the same direction as the vector *P*_2_^a^*P*_3_^a^, pointing upwards. The axis *x*^a^ is perpendicular to the plane (frontal plane) formed by the axis *y*^a^ and the vector *P*_1_^a^*P*_2_^a^, and points towards the front of the human body. The axis *z*^a^ is perpendicular to the plane (sagittal plane) formed by axis *x*^a^ and axis *y*^a^, and in the same direction as vector *P*_1_^a^*P*_2_^a^. In the pelvic kinematic coordinate system *O*_2_^k^(*x*^k^, *y*^k^, *z*^k^, 1) (Figure 1b), the origin *O*_2_^k^ is set at the point (*P*_3_^a^), and the direction of the *z*^k^ axis is in the same direction as the vector *P*_4_^a^*P*_1_^a^. The direction of the *x*^k^ axis is perpendicular to the frontal plane of the human body and points towards the front of the human body; the direction of the *y*^k^ axis is perpendicular to the coronal plane of the human body and points upwards.

Furthermore, based on the coordinates of the pelvic calibration points *P*_1_^a^, *P*_2_^a^, *P*_3_^a^, *P*_4_^a^ in the pelvic anatomical coordinate system and pelvic kinematic coordinate system applied in the anatomical experiment [3], the transformation formula from the pelvic anatomical coordinate system *P*_2_^a^(*x*^a^, *y*^a^, *z*^a^, 1) to the pelvic kinematic coordinate system *O*_2_^k^(*x*^k^, *y*^k^, *z*^k^, 1) is obtained as Equation (2), and its corresponding transition matrix *T*_p_ can be calculated by Formulas (1)–(3). Then, the transition matrix *T*_fe_, *T*_t_, *T*_fo_ of femur, tibia and foot can also be calculated respectively in the same way. Finally, the coordinates of the marks of muscle origins and terminations obtained in the anatomical experiment [30] can be multiplied by the corresponding transition matrix *T*_p_, *T*_fe_, *T*_t_, *T*_fo_ for coordinate transformation, and then their coordinates in the corresponding kinematic coordinate system are obtained, which will be used to calculate the real-time length of the muscles in the kinematic coordinates.

(2) $$\left(x^{k}y^{k}z^{k}\;1\right)=\left(x^{a}\;y^{a}\;z^{a}\;1\right)T_{p}$$

(3) $$T_{p}=\left[\begin{array}{cccc} 0.9133 & -0.4072 & 0 & 0\\ 0.4072 & 0.9133 & 0 & 0\\ 0 & 0 & 1 & 0\\ -0.0646 & -0.0526 & 0 & 1 \end{array}\right]$$

#### 2.3.2. Calculation of the Muscle Forces

Compared with healthy young people, the walking speed of the elderly is generally slower, and the gait posture characteristics are less obvious [29], and corresponding sEMG signal changes weakly, making it more difficult to detect and analyze for processing. In order to make the calculation model of muscle force more suitable for the gait characteristics of elderly people and avoid the involvement of sEMG in the calculation, this paper proposes to use the formula of lower limb muscle fiber force without muscle activation as the formula for calculating muscle active force (1–4), while verifying the formula for calculating muscle passive force (5).

(4) $$f_{l}=\left\{\begin{array}{ll} \exp\left(-{\left[\frac{{\left(\frac{L}{L_{0}}\right)}^{0.963\left(1-\frac{1}{I_{a}}\right)}-1}{0.353\left(1-I_{a}\right)}\right]}^{2}\right), & I_{a}<1\\ \exp\left[-{\left(2.727\cdot \ln\left(\frac{L-L_{0}}{L_{0}}+1\right)\right)}^{2}\right], & I_{a}=1 \end{array}\right.$$

(5) $$f_{p}=0.0195\cdot \exp\left[\left(2.933+\frac{4.911}{I_{a}}\right)\left(\frac{L}{L_{0}}-1\right)\right]$$

where *f_l_* is the normalized muscle active force, *f_p_* is the normalized muscle passive force, *L* is defined as the instantaneous muscle fiber length *L*_0_ is the optimal muscle fiber length and *I_a_* is the muscle architecture index. The muscle architecture index *I_a_* is an important parameter to describe the pennation angle and muscle fiber length. Passive muscle force is only related to the muscle length, so it only becomes prominent and increases when the muscle length is greater than the optimal length of the muscle. Determination of model parameters: The coefficients used in Formulas (4) and (5) are derived from the standard Hill-type muscle model constants optimized for quasi-static conditions [31]. The optimal muscle fiber length (L0) for each subject was determined by scaling the generic values from the OpenSim Gait2392 model. The scaling factor was calculated as the ratio of the subject’s segment length (measured via motion capture) to the generic model’s segment length.

#### 2.3.3. Calculation of Muscle Architecture Index

The muscle structure index is one of the essential key parameters for calculating muscle forces. However, it is difficult to obtain the muscle architecture index based on corpses or live anatomy experiments. Therefore, this article innovatively establishes a multiple regression calculation model to calculate the muscle architecture index (*I_a_*) based on publicly available anatomical data [14,28], where *I_a_* is a parameter that describes the pennation angle and fiber length of muscles. If the pennation angle *x_I_* and the optimum muscle length *y_I_* which represents the exact same physiological parameter as *L*_0_ in Formulas (4) and (5) measured by Delp et al. [29] are set as independent variables, and the muscle architecture index *z_I_* which corresponds to *I_a_* in Formulas (4) and (5) measured by Friederich [32] is set as the dependent variable, the regression calculation model of the muscle structure index can be obtained through multiple regression analysis, as Formula (6):

(6) $$\begin{array}{ll} z_{I}= & 0.1546-5.7662\times 10^{-5}x_{I}+6.6753\times 10^{-4}x_{I}y_{I}\\ & -2.0563\times 10^{-4}{x_{I}}^{2}-1.3233\times 10^{-3}y_{I}+7.6327\times 10^{-4}{y_{I}}^{2} \end{array}$$

where the *r*^2^*_zI_* = 0.98. Since the pennation angle of the source data for the regression calculation model (6) is between 5° and 25°, the model is most suitable for calculating the muscle architecture index with a pennation angle in the range of 5°~25°. Most of the lower limb muscles have a pennation angle larger than 5°, so this regression calculation model defaults the muscles with a pennation angle lower than 5° as parallel muscles with a muscle architecture index of 1.

Finally, based on the obtained coordinates of the marks of muscle origins and terminations, the simplified muscle force calculation Formulas (4) and (5), and the summarized multiple regression calculation model (6) of the muscle architecture index, a simplified Digital Twin Muscle Model (DTMM) can be achieved. Furthermore, based on the anatomical characteristics of the lower limbs, this study explores the force characteristics of the main muscles in each segment of the lower limbs, and nine representative muscles were selected, which are: Gluteus Maximus (Glut_Max1), Tensor Fascia Lata (TFL), Iliacus, Pectineus (Pect), Vastus Intermedius (Vas_Int), Medial Gastrocnemius (Med_Gas), Tibialis Anterior (Tib_Ant), Tibialis Posterior (Tib_Post), and Soleus. After assigning the coordinates of the marks of muscle origins and terminations of each muscle in the kinematic coordinate system to DTSM, a DTSM with the marks of muscle origins and terminations is obtained as shown in Figure 1c. Based on the length of each limb segment and the segment mass—which was determined by multiplying the subject’s total body mass by standard anthropometric proportional coefficients [30], the DTSM with the marks of muscle origins and terminations were scaled to obtain the DTMSM that matched the characteristics of each subject respectively. Finally, the kinematic data and ground reaction force of each subject were assigned to the corresponding DTMSM in OpenSim for inverse kinematics processing, then real-time joint angles, muscle fiber lengths, and muscle forces were calculated based on the muscle architecture index regression calculation model (6) and muscle force calculation Formulas (4) and (5). Thus, the process of non-invasive dynamic evaluation of lower limb muscle forces in elderly and stroke patients has been achieved with a final summarized non-invasive simplified muscle force estimation model (**NSMFEM**).

## 3. Result

By analyzing the output files from the gait movement collection experiments, kinematic and mechanical data of each joint of all the twelve elderly subjects were obtained. During the entire process of muscle exertion, the muscles first passively stretch accompanied by an increase in length, and then actively contract accompanied by a decrease in length. During the process of muscle length shortening, active force is generated, so there is always a cycle offset between the peak length of the muscle and the peak force of the active force. According to Formula (4) presented by Kaufman et al. [14], the muscle active force is calculated. Then the cycle offset between the calculated muscle active force and the corresponding referenced muscle force during human movement under different experimental conditions are shown in Table 1. The cycle offset values presented in Table 1 represent the electromechanical delay and the phase shift between peak muscle length and peak active force. These values were empirically determined from the reference dataset to correct for the temporal lag inherent in kinematic-driven force estimation. The calculated muscle force is then periodically compensated based on the cycle offset values to get the final estimated real-time muscle forces of the nine muscles of the twelve elderly subjects.

### 3.1. Calculation Results of the Muscle Active Forces at Different Walking Speeds

**Figure 2:**
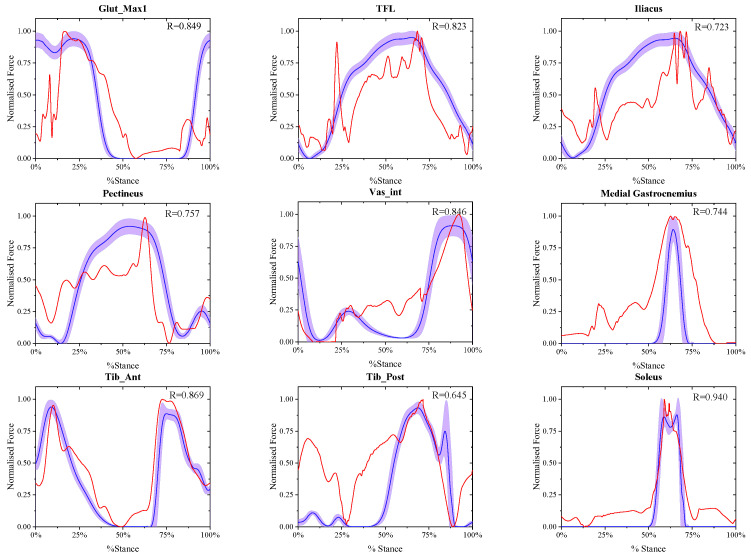
The comparison between the normalized final estimated and the referenced real-time muscle active forces of nine muscles at X-slow walking speed.

**Figure 3:**
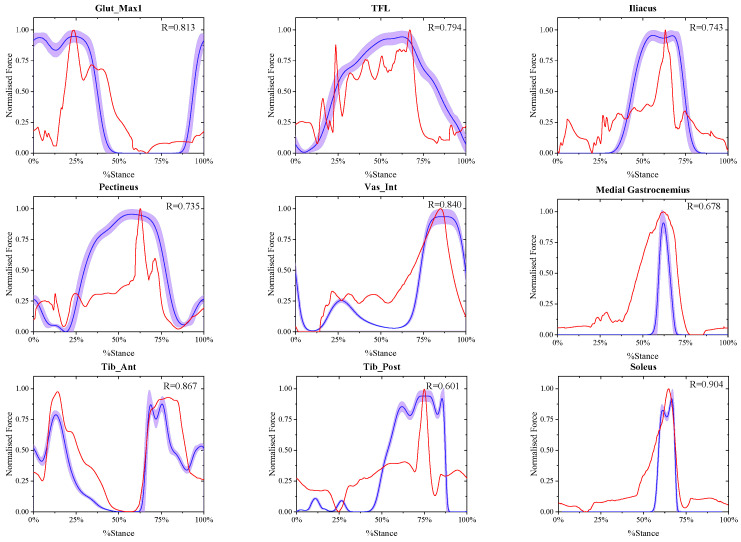
The comparison between the normalized final estimated and the referenced real-time muscle active forces of nine muscles at slow walking speed.

**Figure 4:**
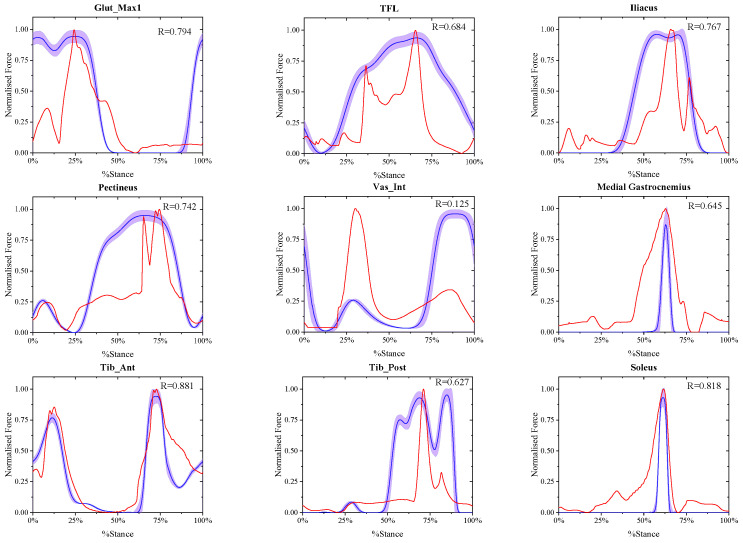
The comparison between the normalized final estimated and the referenced real-time muscle active forces of nine muscles at free walking speed.

**Figure 5:**
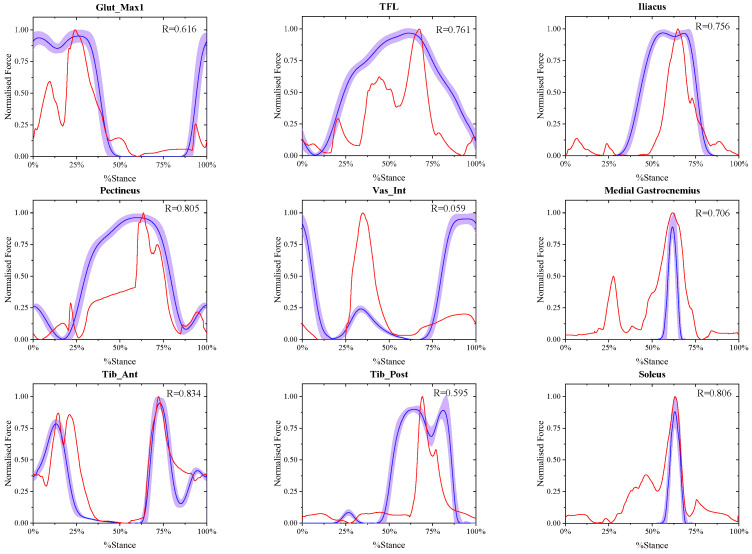
The comparison between the normalized final estimated and the referenced real-time muscle active forces of nine muscles at R-fast walking speed.

The comparison between the final estimated real-time muscle active forces in experiment A and referenced muscle active forces presented by May Q. Liu [33] at the same walking speed of X-slow, slow, free and R-fast is shown in Figure 2, Figure 3, Figure 4 and Figure 5, where in each window the blue solid curve is the mean estimated real-time muscle active force of the same muscle normalized from the eight ESND with the standard deviation as the error band of the purple area, and the red solid curve is the muscle active force normalized from the open-source data presented by May Q. Liu [33] as the reference data, and the correlation coefficient between them was also shown in each windows.

### 3.2. Calculation Results of the Muscle Passive Forces at Different Walking Speeds

**Figure 6:**
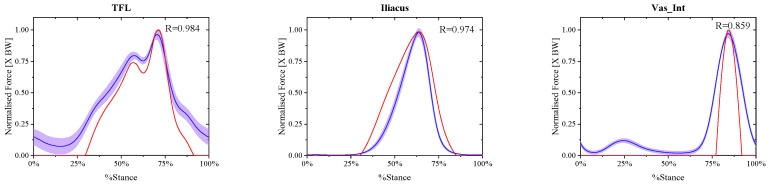
The comparison between the normalized calculated muscle passive forces and the referenced muscle passive forces of three muscles at free walking speed.

The comparison between the final real-time muscle passive forces calculated by Formulas (1)–(5) in experiment A and referenced muscle passive forces presented by May Q. Liu at the same free walking speed is shown in Figure 6. Compared to muscle active force, the magnitude of muscle passive force is only related to the length of muscle fibers [32], so only the three muscles with passive forces among the nine detected muscles were shown in Figure 6. And in each window the blue solid curve is the mean real-time muscle passive force of the same muscle normalized from the eight ESND with the standard deviation as the error band of the purple area, and the red solid curve is the muscle passive force normalized from the open-source data presented by May Q. Liu as the reference data, and the correlation coefficient between them was also shown in each windows.

### 3.3. Calculation Results of Muscle Active Forces on Both Sides of the Lower Limbs in Elderly Hemiplegic Patients

**Figure 7:**
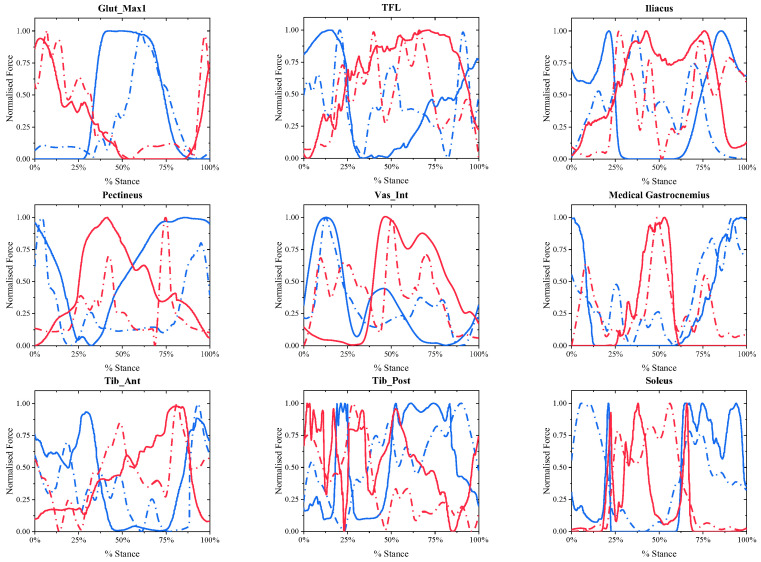
Normalized muscle active forces of 9 muscles under pathological gait conditions of the 68 year old female patient compared with the referenced muscle active forces calculated by OpenSim.

A 68 year old female patient with right hemiplegia after 20 months of stroke onset out of the four ESP in Experiment B was selected to show nine calculated muscle active forces with cycle compensation. Then the calculated muscle active forces of the patient were normalized and compared with the normalized muscle active forces of the corresponding muscles calculated by CMC in OpenSim under the same conditions as the referenced muscle active forces, as shown in Figure 7, where the blue and red solid lines represent the calculated muscle active forces in the left leg and right leg of the patient, and the blue and red dashed lines represent the corresponding referenced muscle active forces.

## 4. Discussion

### 4.1. Analysis of the Active and Passive Muscle Forces of the Eight ESND Under Non-Pathological Gait

**Figure 8:**
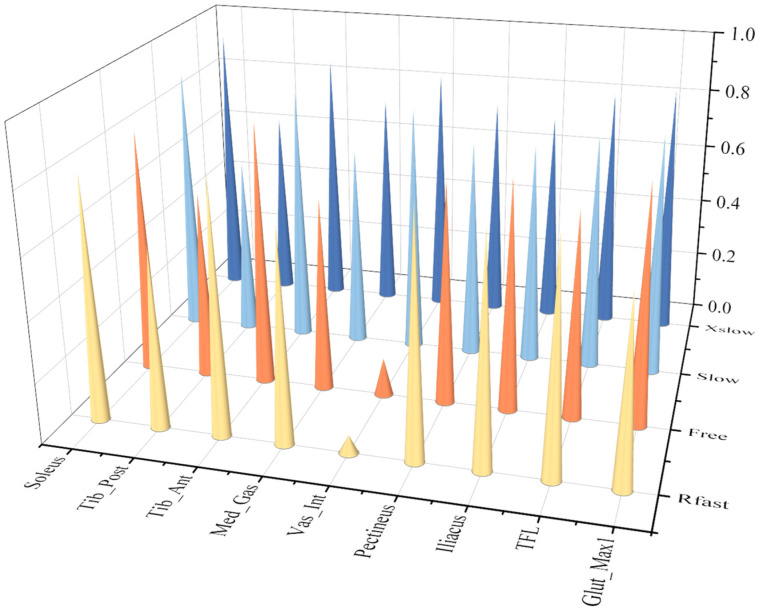
Correlation coefficient graph of nine active muscle forces of the eight ESND under four different speeds of gaits (The vertical axis represents the Pearson Correlation Coefficient (range: 0.0–1.0). The speed labels on the right axis correspond to: X-slow (Extremely slow gait), Slow (Slow gait), Free (Free gait), and R-fast (Fast gait).).

Nine calculated active and passive muscle forces of the eight ESND under four different speeds of gaits were normalized and averaged, and then conducted a correlation analysis with the corresponding reference muscle forces calculated by OpenSim based on the Pearson correlation model. The muscles with muscle passive force are TFL, Iliacus and Vas_Int, and their correlation coefficients are 0.984, 0.974 and 0.859, respectively. Because muscle passive force is only related to the muscle fiber length, the correlation coefficients of the passive forces are all within the range of strong correlation, as shown in Figure 8.

The correlation coefficients of all the muscle active forces are shown in Figure 8, where the correlation coefficients of TFL, Iliacus, Pectineus, Tib-Ant, and Soleus show strong correlation at any speeds, while the other muscles, Glut-Max1 and Vas-Int, only show strong correlation at extremely slow and slow speeds. In addition, the results show that “Med_Gas and Tib_Post” are moderately correlated with the reference data under various working conditions, which reflects the limitations of the muscle force calculation model in this paper. As the speed increases, the correlation decreases significantly. This is mainly because as the walking speed increases, the influence of the acceleration of each limb segment on muscle force gradually increases, and the error of calculating muscle force solely based on muscle fiber length also gradually increases.

Consequently, the NSMFEM presented in this paper has higher accuracy under extremely slow and slow working conditions. As the speed increases, its accuracy begins to decline. Moreover, the muscle force calculation method expected to be established in this paper is applicable to the elderly and rehabilitation patients under slow walking conditions. Only nine representative lower limb muscles were collected during the experimental verification, while the muscle force data of the control group were adult multi-step speed data calculated by May Q. Liu [33]. As a result, the correlation between the muscle force calculation results of “Vas_Int” and “Tib_Post” muscles and the reference data is low.

### 4.2. Correlation of Results for Stroke Gait

**Figure 9:**
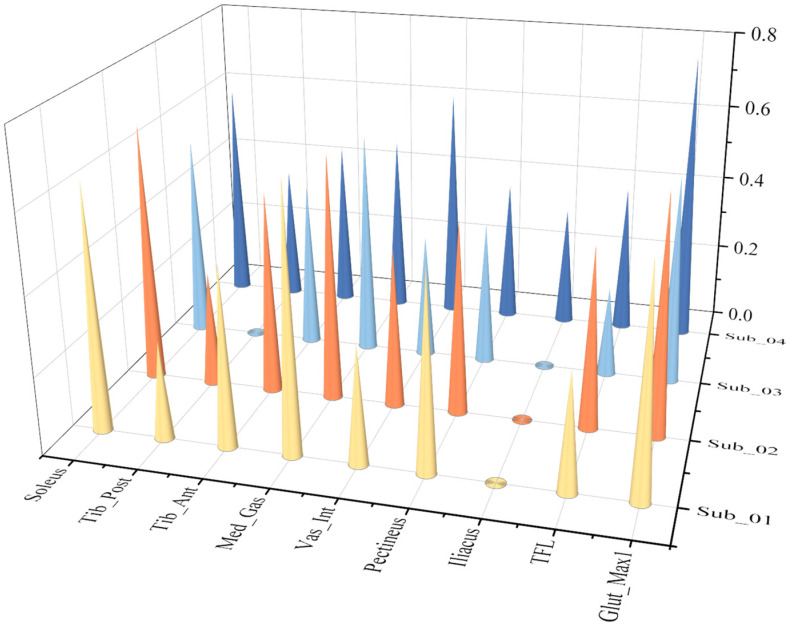
Correlation coefficient graph of active force on the non-affected side under pathological gait (the vertical axis represents the Spearman correlation coefficient (range: 0.0–1.0)).

**Figure 10:**
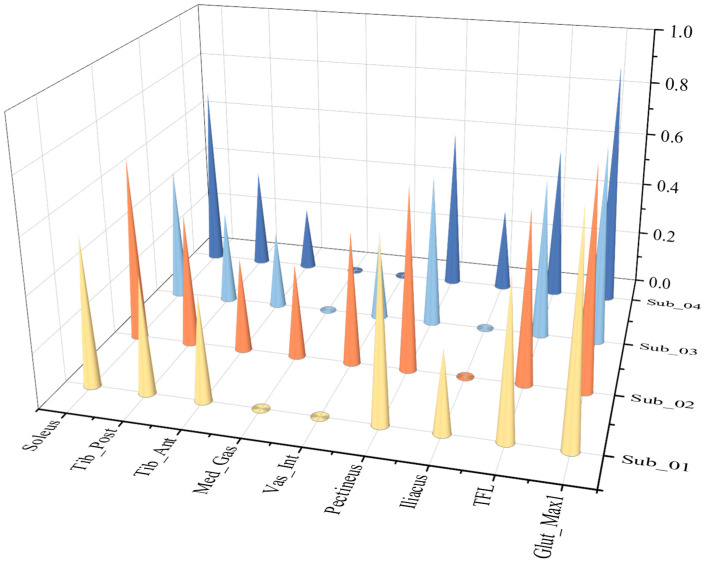
Correlation coefficient graph of active force on the affected side under stroke gait. The vertical axis represents the Spearman correlation coefficient (range: 0.0–1.0).

In the correlation analysis of pathological gait results, due to abnormal gait movements, there is a large deviation in the extreme values of muscle force in muscle force calculation. Therefore, the correlation coefficient calculated by the Pearson correlation coefficient model is generally low, and linear correlation analysis is not suitable for analysis under this working condition. The Spearman correlation coefficient was used for analysis. Correlation analysis was performed between the muscle active forces experimental data of **the four ESPs** and their respective reference muscle force data. Figure 9 and Figure 10 compare the correlation values of 9 muscle active forces among **the four ESPs.**

In addition to the slow walking speed, the elderly are also prone to pathological gait affected by disease. The observed heterogeneity in the correlation results likely reflects the complex neuro-motor adaptations inherent to stroke pathology, rather than model inconsistency alone. Post-stroke gait is characterized by altered muscle synergies and compensatory strategies. As noted in systematic reviews on gait rehabilitation [34], gait and postural stability are highly sensitive to specific interventions and neuro-motor states. Therefore, the NSMFEM’s ability to capture these kinematic variations suggests it could serve as a valuable tool for monitoring functional recovery strategies. As explicitly shown in Figure 7, the proposed NSMFEM exhibits noticeable predictive deviations from the OpenSim reference data for several muscles during post-stroke walking. Unlike normal gait, where muscle force changes relatively smoothly and correlates well with kinematics, stroke gait is heavily characterized by impaired neuromuscular control, such as spasticity, abnormal synergies, and co-contraction. Because our simplified model relies solely on kinematics, it inherently struggles to capture these complex, irregular muscle activation patterns without the integration of electromyographic (sEMG) data. Due to the weakening of the muscle force on the affected side, the gait compensation phenomenon of asymmetrical force on both the healthy side and the affected side of the body will appear in the elderly patients with stroke. In the experimental results of pathological gait Figure 7, a strong correlation was observed between the calculated results and the reference data of the three muscles namely: “Glut-Max1, Med_Gas, Soleus” on the healthy side. Due to the influence of the disease, in the calculation of the healthy side muscles of 4-ESTS, the correlation coefficients between the Iliacus of Sub_01, Sub_02, and Sub_03, as well as the Tib_Sost of Sub_03, and their corresponding reference data are almost zero, indicating no correlation. The other muscles show a medium to high degree of correlation.

In the calculation of muscles on the affected side of 4-ESTS, there was almost no correlation between the calculation results and reference data for the Iliacus muscles of Sub_02 and Sub_03, the Vas_Int muscles of Sub_01 and Sub_04, and the Med_Gas muscles of Sub_01, Sub_03, and Sub_04. However, strong correlation was observed between the calculation results and reference data for the Glut_Max1, TFL, Pectineus, and Soleus muscles. The remaining muscles exhibited moderate correlation. Due to motor deformities and muscle atrophy in the affected limb, hemiplegic subjects exhibit a significantly higher number of muscles with no correlation between calculated muscle force values and reference data compared to the unaffected side. To further evaluate the actively recruited muscles, an exploratory subset analysis was conducted by temporarily excluding muscles that failed to demonstrate correlation. Assuming these specific discrepancies arise from profound pathological spasticity or signal-to-noise limitations inherent to hemiplegic stroke gait, they were excluded from this subset. Within this specific subset, the mathematical average of the correlation coefficients on the affected side was observed to be higher than that on the healthy side. This is due to the influence of gait compensation phenomena, resulting in significant compensatory muscle activation on the unaffected side. Under the premise of isometric contraction, the muscle activation on the unaffected side of hemiplegic subjects is significantly greater than that of normal elderly individuals, leading to significantly better correlation of Glut_Max1, TFL, and Pectineus muscles on the affected side compared to the healthy side. Beyond purely technical validation, these specific results—particularly the predictive deviations and near-zero correlations observed in post-stroke subjects (Figure 7)—provide critical new insights into the field of muscle force simulation and biomimetics. While traditional musculoskeletal simulations focus heavily on minimizing errors in healthy cohorts, our study leverages these very discrepancies to offer a meaningful contribution. Specifically, the deviations between our kinematic-driven predictions and the complex pathological reality serve as a quantitative metric for “neuromuscular decoupling.” In the context of rehabilitation engineering, these quantified differences represent the exact magnitude of compensatory torque that an intelligent exoskeleton controller must supply to bridge the gap between a hemiplegic patient’s impaired muscle output and normal physiological requirements. Consequently, this approach transitions simulation results from purely descriptive biomechanical data to prescriptive design parameters for real-time bionic device control. These findings indicate that, under the specific conditions tested in this study, the proposed model captures key force variations for certain functional muscles in slow gait even without activation inputs. Therefore, while this calculation method demonstrates reasonable estimation capabilities for specific muscle groups in rehabilitation patients, broader physiological generalizations regarding the overall influence of muscle activation during slow walking should be avoided.

### 4.3. Other Influencing Factors

During walking, the lower limb muscles are in a state of dynamic contractions, with the length of muscle fibers changing in real-time. At this point, the length of muscle fibers is an important factor affecting the magnitude of muscle force. At the same time, the force exerted by muscles is also influenced by muscle activation. To estimate the lower limb muscle force during gait walking, EMG is often used as a direct measure to calculate muscle activation. KFM takes the product of muscle activation and muscle fiber force as the normalized muscle force.

**Figure 11:**
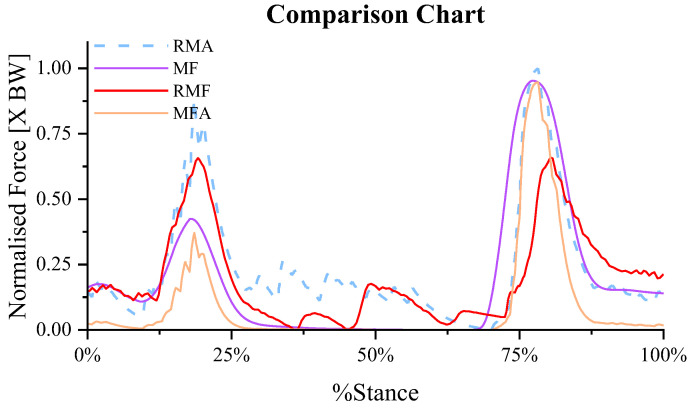
Comparison of muscle force and muscle activation.

Taking the Tib_Ant of an adult with a height of 1.8m as an example, the relationship between muscle force and muscle activation with or without the participation of sEMG was analyzed as shown in Figure 11, where the data of muscle activation (RMA) and muscle force (RMF) calculated by OpenSim were taken as reference data. The calculation result of Formula (1) is taken as muscle force data (MF) without sEMG participation, and the product of muscle activation and Formula (1) is taken as muscle force data (MFA) with sEMG participation. After calculation, the correlation coefficient between MF and RMA is 0.827, and the correlation coefficient between MF and RMF is 0.653 without considering the influence of activation. When considering the influence of activation, the correlation coefficient between MFA and RMA was 0.841, and the correlation coefficient between RMF and MFA was 0.672. It indicates that during slow gait movement within our specific computational framework, the muscle force predictions align closely with reference data even without explicit muscle activation inputs. As a result, even when muscle activation is not considered in the calculation of muscle force and only muscle fiber length is used, the obtained results still exhibit a strong correlation with those of the control group. This suggests that muscle activation parameters have a relatively small impact on the predictive accuracy of this specific model during slow gait. However, this model-specific observation should not be broadly generalized to conclude that muscle activation has minimal physiological influence during slow human walking. Therefore, in the case of error tolerance, we presents a NSMFEM that can accurately calculate the muscle force without using the muscle activation.

### 4.4. Limitations and Broader Applicability

During the geometric scaling and measurement process, the calibration of muscle origin and insertion points is inherently affected by subject-specific anatomical variations and musculoskeletal scaling errors [3], which can lead to a reduced correlation between the calculated results and reference data. Secondly, the muscle structure index regression calculation model in this paper is based on measurement data from Delp [29] and Kaufman [14]. However, due to the limitations of experimental measurement techniques at that time, the regression model suffered from issues such as large measurement errors in muscle data and a small sample size of basic data, resulting in distorted calculation results when the muscles are parallel muscles. Most muscle pennate angles are located between 5° and 25°. In this paper, muscles with pennate angles less than 5° are equivalent to parallel muscles for calculation. Under current conditions, this paper omits the involvement of electromyographic signals based on Kaufman’s muscle force formula [14], and the calculated results of NSMFEM achieve a significant strong correlation with reference data [35]. (Crucially, we acknowledge that the application of this model to stroke patients in the current study is strictly exploratory. A sample size of four post-stroke subjects is insufficient to reliably calculate generalized model coefficients or fully validate its clinical efficacy. Furthermore, stroke pathology encompasses various types and lesion locations, leading to highly heterogeneous degrees of muscle paresis and locomotion impairment. The current model does not account for these sub-classifications or the severe spasticity that uncouples muscle length from active force generation. Furthermore, the shift towards sensor-based, quantitative metrics aligns with recent calls for novel assessment methods in broader wellness and pediatric research. Nevertheless, we acknowledge that referencing this literature contextualizes the clinical relevance of our objectives, but does not inherently validate the NSMFEM methodology itself. While current results focus on muscle force, future applications could integrate these kinematic estimates with quantitative balance measures, such as center of pressure sway analysis, to provide a comprehensive assessment of post-stroke stability. This reinforces the legitimacy of non-traditional quantitative approaches beyond standard clinical tools.

## 5. Conclusions

This study presents an NSMFEM for estimating lower limb muscle forces in the elderly and stroke patients during slow walking. The model, based solely on kinematics and muscle length, computes active and passive force profiles. Validation across multiple subjects and walking speeds showed high correlation with established models at slow speeds, with accuracy declining as speed increases due to the neglected influence of activation. In hemiplegic gait, the model achieved clinically useful correlations for specific muscle groups. Therefore, within defined error tolerances, NSMFEM is suitable for non-invasive muscle force analysis during slow walking in these populations. This approach supports the growing need for accessible, sensor-based biomechanical metrics in clinical settings. Future work will focus on incorporating activation dynamics to extend the model’s applicability to faster walking and more varied pathological gaits.

## Figures and Tables

**Table 1:** The average cycle offset of each muscle out of the nine representative muscles under four walking speeds performed by each of the eight ESND, and the average cycle offset of each muscle out of the nine representative muscles under the self-selected free walking conditions of the four ESP.

Muscles	ESP (Free) Δcycle (100%)	ESND (R-Fast) Δcycle (100%)	ESND (Free) Δcycle (100%)	ESND (Slow) Δcycle (100%)	ESND (X-Slow) Δcycle (100%)
Glut_Max1	11.2	15	17.49	16.15	16.03
TFL	0	5	1.94	0	0
Iliacus	9.27	1.25	0	0	0
Pectineus	4.76	1.25	1.46	0	0
Vas_Int	30.2	42.5	39.84	7.43	3.11
Med_Gas	3.91	7.5	1	0	0
Tib_Ant	18.4	2.75	3.33	0	3.79
Tib_Post	14.5	11.25	6.32	8.1	0.38
Soleus	0	8.75	1.46	5.43	0.32
Mean Value	10.25	10.58	8.09	4.13	2.63

## Data Availability

The data presented in this study are available upon request from the corresponding author.
